# Effect of Creatine Supplementation on Body Composition and Malnutrition-Inflammation Score in Hemodialysis Patients: An Exploratory 1-Year, Balanced, Double-Blind Design

**DOI:** 10.3390/nu16050615

**Published:** 2024-02-23

**Authors:** Ana Clara B. Marini, Raquel M. Schincaglia, Darren G. Candow, Gustavo D. Pimentel

**Affiliations:** 1 Faculty of Nutrition, Federal University of Goiás, Rua 227, Quadra 68 s/n°, Setor Leste Universitário, Goiania 74605080, Brazil; ac.marini22@gmail.com (A.C.B.M.); raquelms@outlook.com (R.M.S.); 2 Faculty of Kinesiology and Health Studies, University of Regina, Regina, SK S4S 0A2, Canada

**Keywords:** hemodialysis, body composition, creatine, malnutrition

## Abstract

Hemodialysis has a detrimental effect on fat-free mass (FFM) and muscle strength over time. Thus, we aimed to evaluate the effect of creatine supplementation on the body composition and Malnutrition-Inflammation Score (MIS) in patients with chronic kidney disease (CKD) undergoing hemodialysis. An exploratory 1-year balanced, placebo-controlled, and double-blind design was conducted with hemodialysis patients (≥18 years). The creatine group (CG) received 5 g of creatine monohydrate and 5 g of maltodextrin per day and the placebo group (PG) received 10 g of maltodextrin per day. MIS and body composition were analyzed at three time points: pre, intermediate (after 6 months), and post (after 12 months). After 6 months, 60% of patients on creatine experienced an increase in FFM compared to a 36.8% increase for those on placebo. Moreover, 65% of patients on creatine increased their skeletal muscle mass index (SMMI) compared to only 15.8% for those on placebo. Creatine increased intracellular water (ICW) in 60% of patients. MIS did not change after the intervention. In the CG, there was an increase in body weight (*p* = 0.018), FFM (*p* = 0.010), SMMI (*p* = 0.022). CG also increased total body water (pre 35.4 L, post 36.1 L; *p* = 0.008), mainly due to ICW (pre 20.2 L, intermediate 20.7 L, post 21.0 L; *p* = 0.016). Long-term creatine supplementation in hemodialysis patients did not attenuate the MIS, but enhanced FFM and SMMI, which was likely triggered by an increase in ICW.

## 1. Introduction

Chronic Kidney Disease (CKD) is characterized by kidney damage and progressive and irreversible loss of kidney function [1,2] and the changes in body composition (i.e., decrease in fat-free mass) of patients with CKD are influenced by several factors. The decrease in fat-free mass may be the result of an elevated inflammatory state and/or reduction in physical function and capacity, total energy, and protein ingestion in these patients. The decrease in fat-free mass is a predictor of mortality in these individuals and the high prevalence of malnutrition increases morbidity, hospitalization time, and mortality [3,4].

The Malnutrition-Inflammation Score (MIS) is a Subjective Global Assessment (SGA)-based instrument and includes three additional items: body mass index (BMI), serum albumin concentration, and total iron-binding capacity (TIBIC) [5]. In a study published in 2019 by our research group, we observed that 12.6% of hemodialysis patients were malnourished (as assessed using the MIS), which was associated with changes in weight, handgrip strength (HGS), and one-repetition maximum leg extension [6]. Noting the impact of CKD and HD on body composition and prognosis, it is important to evaluate how nutrition can influence the reduction of these consequences.

One of the main measures to control muscle depletion is a protein-energy adjustment [7], however, besides low protein intake, treatment also corroborates negative protein balance. Hendriks and collaborators (2020) observed that between 8 and 15 g of amino acids (AA) are removed from circulation in each HD session [8,9] and that nutritional intervention can reduce negative protein balance [10]. Post et al. (2021) found that during an HD session there is a reduction in arginine, guanidinoacetate, creatine, and creatinine. Low creatine values predicted low muscle mass, hypoalbuminemia, and severe fatigue. Interestingly, creatine removed from the intracellular medium (clearance) to the extracellular medium is removed by the dialysate during HD [11]. Creatine is an organic acid found primarily in skeletal muscle and is acquired through the consumption of red meat and seafood or commercially manufactured dietary supplements or endogenously produced from reactions involving the amino acids glycine, arginine, and methionine in the kidneys and liver [12,13]. Creatine’s primary mechanism of action involves rapid energy delivery through the creatine/ATP/creatine kinase system that occurs in mitochondria. The intramuscular amount of creatine and phosphocreatine, when elevated, can increase the mitochondrial energy content favoring an increase in strength for muscle contraction and muscle mass gain [11,14].

In a study published in 2019, our group found that creatine supplementation for one month could reduce MIS and fat-free mass loss in patients who supplemented with 5 g of creatine monohydrate per day. Comparing the groups, it was noted that the creatine group (CG) showed greater gains in fat-free mass than the placebo group (PG) [15]. Because of the benefits found from creatine supplementation on body composition, we hypothesized that long-term supplementation may contribute to the maintenance of fat-free mass (FFM) and a reduction in MIS. Thus, we aimed to evaluate the effect of creatine monohydrate supplementation on the body composition and MIS in patients with CKD undergoing HD.

## 2. Materials and Methods

### 2.1. Design of Study

This exploratory 1-year, balanced, placebo-controlled, and double-blind design was conducted with patients of both sexes diagnosed with CKD undergoing HD, and older than 18 years. The intervention with creatine was twelve months in duration.

**Figure 1:**
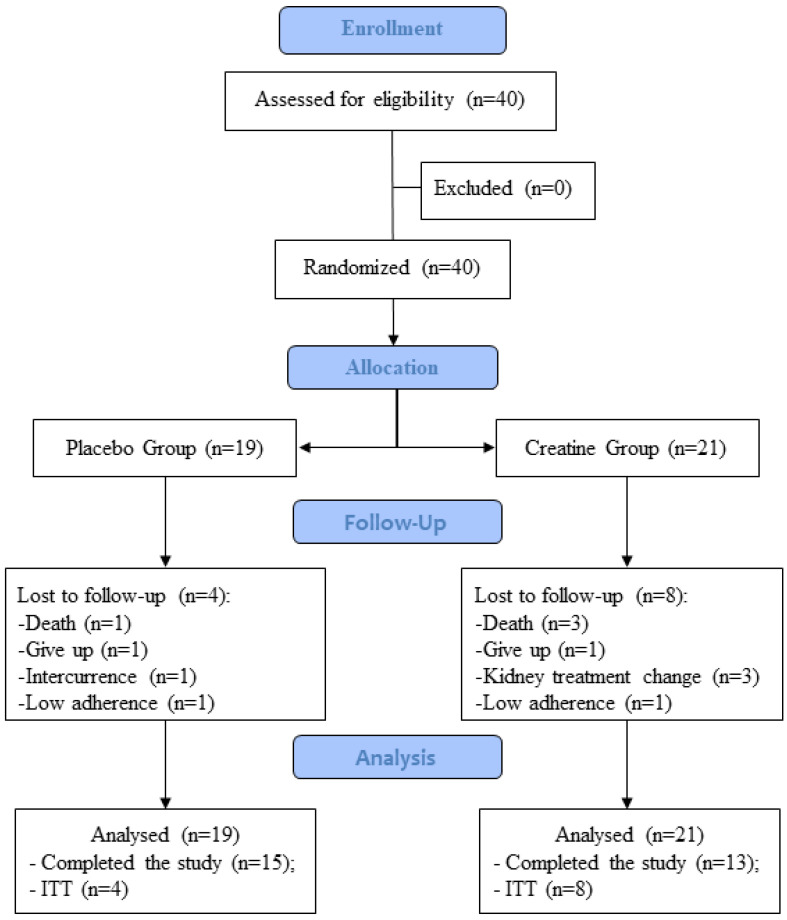
Study flowchart.

After the inclusion of the patients in the study, they were allocated by gender and age (Figure 1). The patients signed the Informed Consent Form approved by the Research Ethics Committee of the Federal University of Goiás, number 4.423.868 (CAAE: 33623220.7.0000.5083 date: 26 November 2020) and registered in Clinical Trial (REBEC code: RBR-55vv9b2 https://ensaiosclinicos.gov.br/rg/RBR-55vv9b2 accessed on 18 August 2022).

### 2.2. Recruitment and Sample Selection

The sample and criteria of inclusion were composed of patients diagnosed with CKD undergoing HD treatment for more than three months in a hemodialysis clinic in Goiânia, GO, Brazil. The Gpower^®^ 3.1 software was used to calculate the sample size [16], in which a significance level of 5% with a statistical power of 80%, the effect size of 0.50, two groups, and two measurements (LBM and MIS) were considered [15], so the study population calculated consisted of 12 patients per group.

Exclusion criteria were patients diagnosed with impaired cognitive function through clinical judgment carried out by the medical team linked to the HD clinic, communication difficulties due to stroke sequelae, hearing impairment identified by the local medical team, amputees, and an HD time of fewer than three months. Patients who had complications (hospitalization, COVID, and other acute illnesses) and who went one week without taking the supplement were excluded.

### 2.3. Experimental Groups

The study was performed with 40 patients divided into two groups balanced by gender and age (Table 1): (1) Placebo Group (PG) composed of 19 patients who received maltodextrin, and (2) Creatine Group (CG) composed of 21 patients who received creatine (Figure 1).

**Figure 2:**
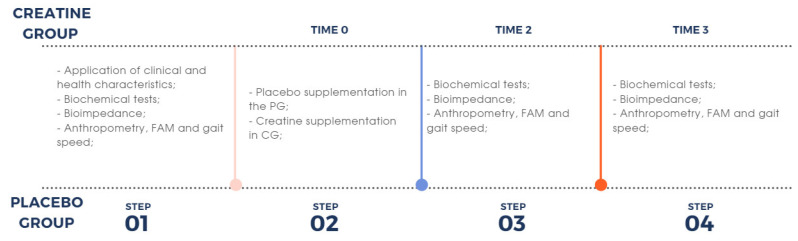
Study design.

The intervention was separated into four steps after the balancing and division of the groups: (1) during the first week of the study, the initial evaluations were performed including MIS, a review of exams in the medical record, and anthropometric measures and body composition (bioimpedance electrical analysis); (2) from the first to the sixth month, the intervention with creatine and the placebo was conducted; (3) during the sixth month of the study, the same parameters as in step 1 were reassessed; and (4) at 12 months, a reassessment of the same parameters as in was performed (Figure 2).

### 2.4. Protocol Supplementation

The blinded intervention was performed with sachets containing either creatine or placebo. These sachets were standardized to avoid any identification of the content by the patients. Because the creatine powder had no taste, whereas maltodextrin was lemon flavored, all doses of creatine were added to maltodextrin. Both creatine and maltodextrin were donated by Probiotica^®^, Supley, Matão, SP, Brazil. Patients were instructed to maintain their eating habits and not to use nutritional supplements.

### 2.5. Malnutrition-Inflammation Score (MIS)

One way to assess the patient quickly and economically is the use of subjective global assessment (SGA). This instrument requires a short period of training, depending essentially on human resources for its use [6]. The MIS developed by Kalantar-Zadeh et al. (2001) is an instrument based on the SGA and includes three additional items: BMI, serum albumin concentrations, and total iron-binding capacity (TIBC) [5].

MIS presents the clinical history and physical and biochemical analysis of the patient. The clinical history consists of addressing aspects such as weight reduction in the last six months, changes in dietary intake, the presence of gastrointestinal symptoms, and functional capacity related to nutritional status. Physical examination includes aspects such as subcutaneous fat loss, muscle loss, the presence of edema resulting from malnutrition, and ascites which have been defined as normal, or mild, moderate, or severe malnutrition. Biochemical parameters include albumin and TIBC. After completing the clinical, physical, and biochemical examinations, the results can range from 1 to 30, and then the classification of the nutritional status is performed. A score ≤ 6 presents normality and a score > 6 presents classification for malnutrition and high MIS [5,15,17,18].

### 2.6. Anthropometric and Body Composition Assessment

Anthropometric data were collected during the intermediate session of the week of HD (2nd session). Weight and height were evaluated by an anthropometric digital scale (Filizola^®^ Brazil). Measurements were performed in duplicate (non-consecutive measurements) after the HD session. The procedures followed the recommendations of Gibson (2005), and the patients were weighed without shoes and wearing light clothes in a standing position in the center of the platform, with feet parallel, arms extended along the body, and head erect, looking forward. For height, the patient was assessed barefoot, with heels together, keeping legs and back straight and arms along the body. Heels and buttocks lightly leaning against the wall and the head held straight [19]. 

In addition, calf circumference was measured using a flexible tape measure. Calf circumference was performed with the individual in the supine position, knees bent at a 90° angle, and heels resting on the bed or chair. The data were collected in duplicate by a trained dietician [20].

Body composition was assessed by a bioimpedance medical device Body Composition Analyzer (Model mBCA 525, Seca, Hamburg, Deutschland, Germany). The device was read in the supine position, using eight disposable electrodes, in the positions of the wrists (between the radius and the ulna) and between the metacarpus of the hands, on the ankles (on the talus), and between the metatarsals of the feet. This was measured after the HD session, and to avoid interference from interdialytic weight gain, it was performed in the middle session of the week (second session of the week). The Skeletal Muscle Mass Index (SMMI) was recorded from bioimpedance data, and it is defined as the appendicular skeletal muscle mass/height² [21,22].

### 2.7. Biochemical Analysis

Assessments were made for serum creatinine (Alkaline Kinetic Picrate), urea (Urease), and phosphorus (Phosphomolybdate) using the Abbott methods. 

The protein equivalent of nitrogen appearance (PNA) value was calculated by the computerized system (Nephrosys) through the formula that uses the Kt/v and serum nitrogen pre-hemodialysis as parameters. Kt/v being a value originated from a calculation used to assess the adequacy of hemodialysis. The method approved by the KDOQI and used in this study is the Daugirdas equation (1996): spKt/V = −ln(R − 0.008 x t) + (4 − 3.5 × R) 0.55 × UF/V where R is pre-urea/post-urea, t is the session duration in hours, −ln is the negative natural logarithm, UF is the weight loss in kilograms, and V is the anthropometric urea volume of distribution in liters [23].

PNA (g/day) = Pre-dialysis serum urea nitrogen

           [36.3 + (5.48) × (Kt/V) + 53.5/Kt/V)] + 0.168

### 2.8. Statistical Analyses

The data were deposited in Microsoft Excel^®^ and transcribed into the software Statistical Package of Social Sciences (SPSS) 21 and G*Power 3.1 for sample calculation and effect size. Descriptive statistics (absolute and relative frequencies, mean, and standard deviation (SV) were used. The continuous variables were tested for normality by the *Shapiro-Wilk* Test. Chi-square and Fisher’s exact test were used to evaluate categorical variables. 

For variables that did not show normality, the Mann–Whitney test was used to assess the differences between the groups (PG Pre vs. CG Pre; PG Intermediate vs. CG Intermediate; PG Post vs. CG Post) and the Friedman test to assess the differences within the groups over the time (Placebo and Creatine). For the variables that showed significance in the Friedman test, the Wilcoxon test was performed to find out which of the moments there was a difference. The level of statistical significance was set at 5% (*p* < 0.05). The effect size was estimated by the Cohen d test from the calculation of differences between groups and between baseline and final moments.

The analyses were performed at three time points: pre, intermediate (after 6 months of intervention), and post (after 12 months of intervention). We still represent the differences between the moments as Time 1 (difference Intermediate vs. Pre), Time 2 (difference Pre vs Post), and Time 3 (difference Post vs. Intermediate).

During the intervention there were sample losses due to several factors as shown in Figure 1, thus causing missing data or dropouts. Those withdrawals cause a reduction in the sample and promote a false estimate of the treatment effect. Thus, the sample was evaluated with the imputation of data (by intention to treat—ITT), a method in which the value of the last result of the individual at the moment that is missing was replicated (last observed carried forward-LOCF).

## 3. Results

### 3.1. Baseline Characteristics and Food Intake

The baseline characteristics of the patients are shown in Table 1. Both groups were similar in sex, age, BMI, hemodialysis time, Kt/v, and life habits.

### 3.2. Malnutrition-Inflammation Score (MIS)

MIS did not change the difference during the intervention (Table 2).

### 3.3. Intention to Treat

**Figure 3:**
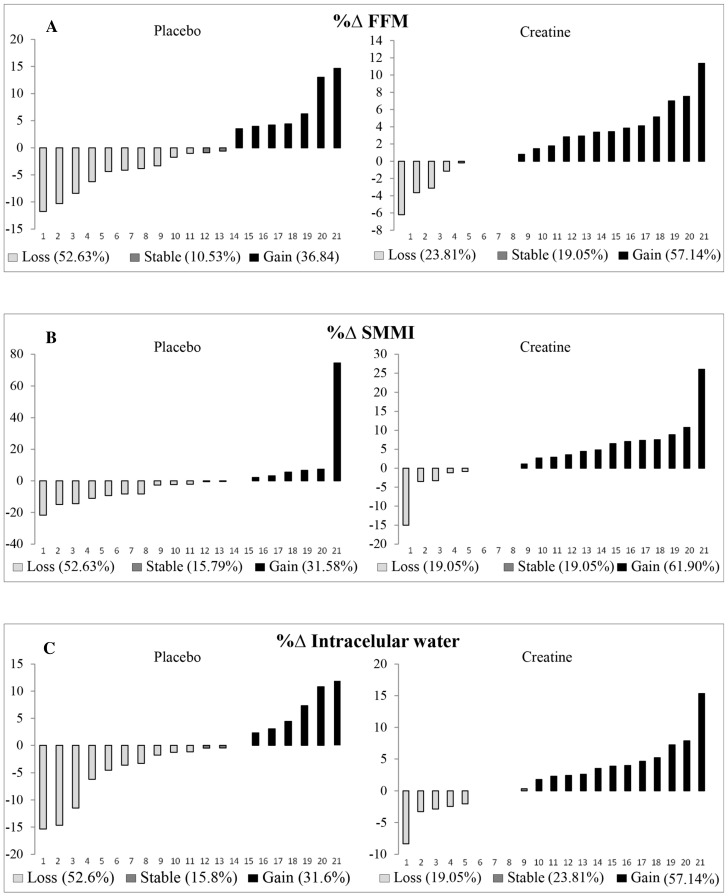
Delta of individual change at Time 1 in %FFM (**A**), %SMMI (**B**), and % intracellular water (**C**). Time 1: Difference between pre and intermediate time points. %∆SMMI: Delta percentage Skeletal Muscle Mass Index; %∆FFM: Delta percentage Fat-Free Mass.

**Figure 4:**
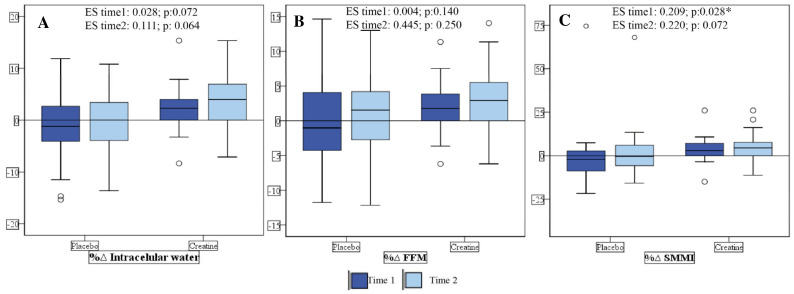
Delta of % change in intracellular water (**A**), Fat-Free Mass (FFM) (**B**), and Skeletal Muscle Mass Index (SMMI) (**C**) in the creatine and placebo groups. ES: Effect size. Time 1: Difference between pre and intermediate time points; Time 2: Difference between pre and post. * *p* < 0.05 was considered as significant.

In the CG there was an increase in dry weight, FFM, and SMMI. The CG also had increased the TBW, mainly due to the intracellular water content (Table 2). Additionally, 60% of patients from the CG increased their FFM and 20% reduced their FFM, whereas in the PG, only 36.8% increased and 52.6% reduced their FFM at Time 1. Moreover, 65% of the patients from the CG had an increase in the SMMI and 20% presented with a reduction, whereas in the PG, only 15.8% had an enhancement and 52.6% a reduction at Time 1. For ICW, 60% of CG patients had an increase and 20% had a decrease, whereas 15.8% of PG patients had an increase and 52.6% had a decrease at Time 1 (Figure 3 and Figure 4).

In the CG, we found an increase in creatinine and post-urea levels at Time 1 and Time 2 of the intervention. In addition, at Time 1 the CG had increased pre-urea levels compared to the PG (Table 1). In the PG, at the intermediary moment, there was a reduction in phosphorus levels, but post-intervention it returned to the pre-intervention levels (5.2 mg/dL); the same occurs in PNA values, with a reduction to 0.9 at the intermediary moment and a return to 1.0 post-intervention (Table 1).

**Figure 5:**
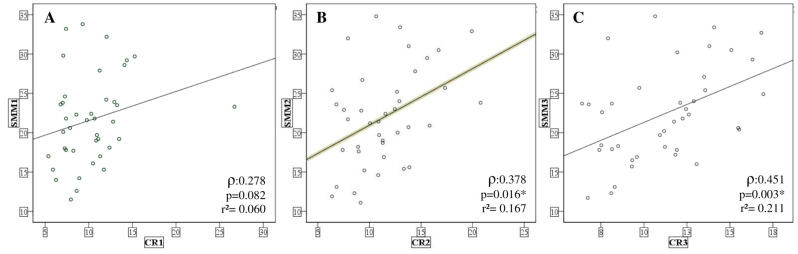
Spearman’s correlation test between SMMI and creatinine. CR: creatinine, SMMI: Skeletal Muscle Mass Index. (**A**) Pre; (**B**) Intermediate; and (**C**) Post; * *p* < 0.05 was considered as significant.

When performing bivariate correlation analysis with Spearman’s test between the SMMI and creatinine levels, we did not find any correlation between the variables at the baseline. However, at the intermediary moment, there is a positive correlation (ρ = 0.378, *p* = 0.016) and the same happens post-intervention (ρ = 0.451, *p* = 0.003), which could suggest that the increase in SMMI values may be due to the enhancement of the creatinine level (Figure 5).

## 4. Discussion

Our study showed that creatine supplementation, compared to placebo, increased SMMI and FFM, which was likely influenced by ICW. These results indirectly support the previous finding where we showed that creatine increased muscle strength when assessed by HGS (+3.2 kg, ES:0.49, *p* = 0.037) and leg-extension 1-RM (+14.2 kg, ES: 0.47, *p* = 0.005) and improved the quality of life-related physical role function (+56.8 points, ES: 1.11, *p* = 0.012) in HD patients [24]. Likewise, Veen et al. (2021) proposed a study protocol design to be performed with HD patients based on a reduced ability to synthesize creatine, losses in dialysis fluid, and insufficient intake due to plant-based dietary recommendations that cause creatine deficiency [25]. Although results for this protocol study are not yet available, and his proposal is with addition of creatine in the dialysis solution, we were able to demonstrate beneficial results on body composition, in particular FFM, using oral creatine supplementation. Therefore, we highlighted that long-term creatine supplementation may be useful and safe for HD patients.

However, creatine had no effect on MIS. We have previously shown that creatine supplementation for one month in adults on HD attenuated MIS (Pre: 5.5 ± 0.7 vs. Post: 3.8 ± 0.4 score, *p* = 0.003) compared to the PG (Pre: 5.7 ± 0.9 vs. Post: 5.3 ± 0.9 score, *p* = 0.317) [15]. We speculate that these contrasting results may be based on the potential initial beneficial effects of creatine which subside over time. In a study carried out in Spain involving 2748 HD patients, was also found that MIS was associated with catheter implantation and use, type of HD, the absence of residual renal function, older age, comorbidities, and being male [26]. We can therefore understand that during the intervention period of the present study, these factors were not modified, however, we did not evaluate these parameters to verify this association.

Among AA and other substances removed during HD Post et al. (2021) found a reduction of 1939 ± 871 μmol in arginine, 37 ± 20 μmol in guanidinoacetate, 719 [399–1070] μmol in creatine, and 15.5 ± 8.4 mmol in creatinine, and even after confounding adjustments, there was an association of low creatine values with higher odds of low muscle mass (*p* = 0.04), low protein intake (*p* = 0.02), hypoalbuminemia (*p* = 0.008), and severe fatigue (*p* = 0.006). However, it was observed that there is a removal of creatine from the intracellular to the extracellular environment, which is removed by the dialysate during HD [11].

Creatine can cause, therefore, an improvement in strength and physical capacity, and when associated with exercises can cause muscle mass gain [27]. These results may have been found because creatine can promote greater muscle glycogen storage and thus glycogen replenishment is important to promote training recovery during periods of intense exercise and can still reduce muscle damage, thus tolerating periods of intense training to a greater degree and promoting muscle mass gain [28].

In the elderly, creatine appears to have the potential to impact muscle mass and function through anabolic, anti-catabolic, and regenerative activities, as well as indirectly impacting muscle function through its temporal and spatial ability to accelerate ATP regeneration [29]. In addition, a study carried out by Gualano et al. (2014), already mentioned above, concluded that creatine supplementation associated with resistance training has better results for muscle mass, however, the use of creatine alone (without resistance training) showed an increase in appendicular muscle mass [30].

In a previous study by our group, it was found that creatine supplementation can attenuate the loss of LBM compared to placebo. After one month of supplementation, 28.6% and 71.4% of the PG patients had LBM loss and remained stable, respectively. On the other hand, in the CG, 14.4% of the patients showed loss of LBM, 42.8% remained stable and 42.8% gained [15]. Likewise, in the present study, we confirmed that long-term supplementation with creatine may be beneficial. In other words, 60% of patients from the CG had increased FFM and 20% had reduced FFM, whereas in the PG, only 36.8% had increased and 52.6% had reduced FFM at Time 1. Moreover, 65% of patients from the CG had an increased SMMI and 20% presented with a reduction, whereas in the PG only 15.8% had an enhancement and 52.6% had a reduction at Time 1. Although in the previous study we did not evaluate hydration status (TBW) and storage compartments of these fluids (ICW and ECW), it is probable that the gain of lean mass is related to the increase in TBW.

There is a divergence in the literature regarding the possibility of creatine supplementation causing an increase in ICW, reflecting the increase in TBW [28]. Creatine is an osmotically active molecule and the creatine cycle is accompanied by the specific, saturable, Na+ and Cl− dependent creatine transporter, responsible for the uptake of creatine through the plasma membrane, which can lead to the accumulation of water to maintain the intracellular osmolarity of the muscle [31]. However, some studies show that due to the activity of sodium-potassium pumps, concentrations would not be affected for a long period [28].

In 2003, Power and collaborators carried out an experimental study with young people of both sexes who practiced resistance exercise. The groups were divided between creatine supplementation (with loading phase) and placebo and after one month of intervention the group that used creatine showed a greater increase in body mass and this increase was probably due to water retention during supplementation. One of the volunteers reported having a maintenance body mass in the last year and gained 4.8 kg in body mass during the first week of supplementation. However, supplementation did not change the distribution of body fluids, being stored equivalently in the intracellular and extracellular compartments [32].

Creatine undergoes continuous, non-enzymatic degradation to creatinine daily, which accounts for 1.6 to 1.7% of the total creatine pool per day [13,31]. Thus, creatine supplementation can cause an increase in creatinine concentrations, which occurred in a previously reported study with creatine supplementation for one month with a loading phase [15]. The same occurred in the present study with a significant increase in creatinine.

The present study presented several positive points: (1) the use of BIA to assess body composition and hydration status, as well as body fluid compartments; (2) the use of PNA to estimate that food consumption that did not influence the results of supplementation; and (3) this is the first study with a long-term creatine supplementation period in HD patients. The main limitations are as follows: (1) we did not assess food intake through dietary food recall; and (2) we did not assess catheter use, type of HD, interdialytic weight gain, and residual renal function; thus, these data must be interpreted with caution.

## 5. Conclusions

In conclusion, long-term creatine supplementation may contribute to FFM and SMMI loss even without exercise. However, it may be related to an increase in intracellular water content. Thus, we believe that further studies should be carried out to investigate how creatine supplementation affects hydration status as well as the effects of creatine combined with exercise.

## Figures and Tables

**Table 1:** Baseline characteristics.

Variables	Placebo (*n* = 19) Mean ± SD	Creatine (*n* = 21) Mean ± SD	*p*
Sex (n) ^a^			
Female	7	8	1.000
Male	12	13	
Age (years) ^b^	52.8 ± 17.2	52.9 ± 15.6	0.998
Body mass index (kg/m²) ^b^	25.0 ± 2.8	25.5 ± 4.3	0.661
Hemodialysis time (months) ^b^	54.0 ± 37.4	37.0 ±24.6	0.096
Kt/v ^b^	1.6 ± 0.3	1.6 ± 0.3	0.694
Creatinine (mg/dL)	11.1 ± 4.3	9.4 ± 2.9	0.140
Urea pre (mg/dL)	116.3 ± 31.3	113.2 ± 30.4	0.759
Urea post (mg/dL)	31.0 ± 11.7	29.9 ± 15.0	0.790
Phosphorus (mg/dL)	5.2 ± 1.7	5.2 ± 1.6	0.934
PNA (g/kg)	1.0 ± 0.2	1.0 ± 0.2	0.547
Physical activity ^a^			
Active	6	9	0.527
Inactive	13	12	
Smoking ^c^			
Smoker	2	2	0.894
Non-smoking	10	13	
Ex-smoker	7	6	
Alcohol intake ^c^			
Yes	5	4	0.712
No	14	17	

^a^ Chi-square. ^b^ *t*-test for equality of means. ^c^ Fisher’s exact test.

**Table 2:** Comparison of crude values after intervention time for MIS, body composition, and biochemical parameters between groups. Intent-to-treat analyses.

Variables	Placebo (*n* = 19)			Creatine (*n* = 21)			^1^ Pre p	^1^ Inter p	^1^ Post p	^2^ PG p	^2^ CG p
	Pre	Intermediate	Post	Pre	Intermediate	Post					
MIS	4.7 ± 2.4	3.8 ± 2.4	4.5 ± 2.4	4.4 ± 3.4	3.9 ± 2.4	4.4 ± 2.4	0.434	0.901	0.061	0.362	0.545
Body composition											
Body weight (kg)	67.5 ± 13.6	67.8 ± 13.0	67.4 ± 12.7	71.4 ± 15.4	72.4 ± 15.7	72.2 ± 16.1	0.629	0.597	0.588	0.167	0.018 *^a^
Handgrip strength (kg)	29.58 ± 7.72	29.59 ± 7.32	31.13 ± 7.64	28.91 ± 9.65	30.91 ± 11.62	30.43 ± 11.29	0.745	0.839	0.655	0.223	0.291
Gait speed (m/s)	1.02 ± 0.95	0.90 ± 0.27	0.82 ± 0.24	1.00 ± 0.22	0.94 ± 0.16	0.90 ± 0.17	0.087	0.859	0.390	0.338	0.150
Fat free mass (kg)	47.83 ± 9.28	47.98 ± 11.57	48.45 ± 11.50	47.97 ± 10.02	48.95 ± 10.61	49.50 ± 10.52	0.989	0.871	0.695	0.399	0.010 *^ab^
SMMI (kg)	21.54 ± 5.36	21.42 ± 5.71	21.88 ± 5.58	21.70 ± 6.01	22.55 ± 6.77	22.85 ± 6.42	0.924	0.755	0.695	0.389	0.022 *^ab^
TBW (L)	35.17 ± 6.80	35.39 ± 8.54	35.70 ± 8.50	35.45 ± 7.31	36.12 ± 7.64	36.51 ± 7.62	0.935	0.715	0.645	0.453	0.008 *^ab^
TBW (%)	52.83 ± 7.18	52.00 ± 8.09	52.82 ± 7.57	50.23 ± 7.48	50.22 ± 7.67	51.00 ± 8.41	0.303	0.569	0.473	0.882	0.750
ECW (L)	14.81 ± 3.18	15.19 ± 4.14	15.28 ± 4.15	15.21 ± 3.00	15.38 ± 2.78	15.51 ± 2.93	0.635	0.569	0.616	0.945	0.066
ECW (%)	22.16 ± 2.96	22.30 ± 4.03	22.53 ± 3.67	21.60 ± 3.31	21.50 ± 3.33	21.76 ± 3.74	0.448	0.850	0.542	0.986	0.901
ECW/TBW (%)	42.06 ± 3.03	42.85 ± 3.19	42.67 ± 3.48	43.12 ± 3.36	42.97 ± 4.19	42.76 ± 3.41	0.329	0.725	0.924	0.691	0.481
ICW (L)	20.36 ± 3.93	20.20 ± 4.66	20.42 ± 4.66	20.24 ± 4.60	20.75 ± 5.23	21.00 ± 5.00	0.675	0.924	0.860	0.476	0.016 *^ab^
ICW (%)	30.67 ± 4.84	29.70 ± 4.68	30.28 ± 4.74	28.63 ± 4.93	28.71 ± 5.37	29.24 ± 5.44	0.223	0.579	0.379	0.128	0.427
Biochemical parameters											
Creatinine (mg/dL)	11.15 ± 4.36	11.42 ± 3.47	10.94 ± 2.60	9.40 ± 2.92	11.50 ± 3.69	11.40 ± 3.39	0.203	0.914	0.776	0.135	0.001 *^ab^
Urea pre (mg/dL)	116.3± 31.3	102.6 ± 23.3	118.8 ± 37.2	113.2 ± 30.4	121.1 ± 34.5	124.6 ± 31.5	0.957	0.040 *	0.432	0.082	0.220
Urea post (mg/dL)	31.0 ± 11.7	26.6 ± 7.4	31.2 ± 9.3	29.9 ± 15.0	32.3 ± 14.0	34.7 ± 12.7	0.569	0.212	0.481	0.135	0.003 *^b^
Phosphorus (mg/dL)	5.3 ±1.8	4.8 ± 1.4	5.2 ± 1.6	5.2 ± 1.6	5.1 ± 1.4	5.6 ± 1.6	0.935	0.626	0.597	0.037 *	0.257
PNA (g/kg)	1.05 ± 0.27	0.95 ± 0.21	1.07 ± 0.33	1.00 ± 0.20	1.09 ± 0.29	1.06 ± 0.27	0.935	0.067	0.850	0.014 *^ac^	0.137

CG: creatine group; ECW: extracellular water; ICW: intracellular water; MIS: Malnutrition-Inflammatory Score; PG: placebo group; PNA: protein equivalent of nitrogen appearance; SMMI: Skeletal Muscle Mass Index; TBW: total body water; ^1^ Mann–Whitney Test comparing the groups for each crude value separately (PG Pre vs. CG Pre; PG Intermediate vs. CG Intermediate; PG Post vs. CG Post); ^2^ Friedman Test comparing the crude values for each group separately (Placebo and Creatine); * *p* < 0.05 was considered as significant; ^a^ Time 1: Difference between Pre and Intermediate Wilcoxon Signed Ranks Test; ^b^ Time 2: Difference between Pre and Post Wilcoxon Signed Ranks Test; ^c^ Time 3: Difference between Intermediate and Post Wilcoxon Signed Ranks Test.

## Data Availability

The datasets generated and/or analyzed during the current study are available from the corresponding author on reasonable request.
